# Global Trends in Sun Safety and Skin Cancer Prevention Education Among Adolescents: A Bibliometric Analysis

**DOI:** 10.7759/cureus.114811

**Published:** 2026-08-19

**Authors:** Madison Sigler, Alexa Kelman, Claudia Loret de Mola, Nadiya A Persaud, Linda Brecher

**Affiliations:** 1 Research, Orlando College of Osteopathic Medicine, Winter Garden, USA; 2 Public Health, University of South Florida, Tampa, USA; 3 Specialty Care, Orlando College of Osteopathic Medicine, Winter Garden, USA

**Keywords:** adolescents, adolescent sun safety, bibliometric analysis, preventive dermatology, skin cancer, skin cancer prevention, sun protection education, ultraviolet radiation

## Abstract

Adolescent sun safety education is an important area of prevention research, particularly as sun-protective behaviors established during youth may influence long-term skin health. This bibliometric review examined the scholarly landscape of adolescent sun safety and skin cancer prevention education, with attention to publication trends, collaboration patterns, and major research themes. Records were identified through a PubMed search and analyzed using bibliometric mapping software to evaluate co-authorship networks, institutional collaboration, and keyword co-occurrence patterns. The analysis demonstrated a growing body of literature with increasing attention to behavioral, educational, school-based, and digital approaches to sun safety promotion. Authorship and institutional networks suggested that the field includes several active research clusters but remains relatively fragmented, with opportunities for stronger collaboration across public health, clinical, and educational disciplines. Keyword patterns reflected continued emphasis on adolescent knowledge, ultraviolet exposure, sunscreen use, prevention behaviors, and intervention development. Emerging themes also suggested increasing interest in mobile health and technology-supported strategies. However, limited representation of equity-focused terminology indicates a need for more inclusive research addressing diverse adolescent populations. Overall, this review highlights the evolution of adolescent sun safety education research and identifies opportunities to strengthen future work through interdisciplinary collaboration, standardized outcome measures, scalable interventions, and greater attention to health equity.

## Introduction and background

Skin cancer is one of the most common and preventable cancers in the United States, making preventive education essential for lowering incidence and mortality. Despite this, the U.S. Surgeon General has identified skin cancer as a major public health concern due to its rising incidence and largely preventable risk factors [1]. Nearly five million people in the United States are treated for skin cancer each year, costing approximately $8.1 billion annually and placing a significant burden on the healthcare system [1]. Ultraviolet (UV) radiation exposure is the most important modifiable risk factor, and exposure during childhood and adolescence plays a critical role in determining lifetime skin cancer risk [1]. While genetic factors, such as fair skin, light-colored eyes, the presence of nevi, and family history, are non-modifiable, behavioral factors, such as preventing sunburns, offer a clear path for intervention [2]. In pediatric populations, melanoma risk factors are less established but may include immunosuppression, a history of retinoblastoma, giant congenital nevi, and phenotypic traits such as freckling and poor tanning ability [3].

Importantly, many modifiable risk factors begin early in life. UV exposure during youth leads to cumulative skin damage over time, increasing the risk of developing melanoma and nonmelanoma skin cancers later in adulthood, including basal cell carcinoma, which contributes significantly to skin cancer morbidity and healthcare costs [4]. Melanoma is also one of the most common cancers among individuals under 30, particularly young women [5]. Longitudinal data from 1973 through 2009 identified a steady 2% annual increase in childhood and adolescent melanoma incidence in the United States, predominantly affecting females and older adolescents [6]. While contemporary public health efforts aim to curb these trends, preventing severe UV damage during adolescence remains essential for reducing long-term skin cancer risk. Gender-specific trends also emerge in anatomical distribution. A U.S. cohort study spanning 1973-2009 found that melanoma in boys more commonly occurred on the face and trunk, whereas in girls, it was more frequently found on the lower extremities and hips [6]. Despite the importance of prevention, fewer than one-third of U.S. youth consistently use effective sun protection measures [7].

Adolescence represents a consequential and time-sensitive window for behavioral intervention. Promoting sun safety during this period through clinical counseling, public health campaigns, and digital media platforms can lead to long-term reductions in skin cancer risk. Schools, in particular, provide a unique environment for these strategies. The benefits of promoting sun safety in schools include improving protective behaviors through both classroom education and policy changes, while also allowing schools to model healthy practices for students, families, and the broader community [4]. However, these efforts are often isolated. For maximum impact, school-based initiatives should be reinforced by healthcare provider guidance and home-based family involvement, creating a consistent message across the adolescent’s social ecosystem [8]. In addition, several barriers exist, including school policies that restrict protective items such as hats and sunglasses or limit sunscreen use by requiring nurse application or physician prescriptions [9]. Addressing these gaps in education and policy is essential for improving sun protection behaviors and ultimately reducing the long-term burden of skin cancer.

Prior work in this area has largely taken the form of systematic reviews and meta-analyses evaluating the clinical efficacy of individual educational interventions, or narrative summaries of behavioral risk factors. No bibliometric analysis has yet mapped the scholarly landscape of adolescent sun safety and skin cancer prevention education, leaving open questions about which authors, institutions, and countries are driving research output, whether collaboration spans clinical, public health, and educational disciplines, and how the field's thematic priorities have shifted over the past four decades. This study addresses that gap by asking three questions: how has publication volume changed over time, which authors and institutions form the field's core collaborative structure, and what thematic patterns, including gaps in representation, emerge from keyword co-occurrence across the literature. To address these questions, this study focuses on adolescents aged 13-18 years, consistent with PubMed’s standard adolescent indexing category and the developmental period during which parental oversight of sun protection declines, personal autonomy increases, and behaviors such as intentional sunbathing and indoor tanning often emerge. Broader international and clinical definitions of adolescence may extend beyond this age range. Unlike traditional systematic reviews, which assess the clinical efficacy of individual interventions, bibliometric methods can characterize broader research paradigms, knowledge translation across fields, and institutional collaboration networks [10].

Bibliometric analysis is a quantitative approach that uses publication metadata, such as authorship, institutional affiliation, and keyword frequency, to map the structure and evolution of a research field. It is commonly visualized through network diagrams in which nodes represent entities, such as authors, institutions, or keywords, and connecting lines represent co-occurrence or collaboration relationships, making it well suited to characterizing how a field's collaborative and thematic structure has developed over time. Accordingly, we analyzed publication trends, co-authorship and institutional networks, and keyword co-occurrence patterns to capture the field’s macro-level structure. Since the search was limited to English-language publications indexed in PubMed, the findings represent the international authors and institutions captured within this database rather than an exhaustive multilingual, multidatabase census of the literature.

## Review

Methods

Objectives

The paper provides a bibliometric overview of global research output concerning sun safety and skin cancer prevention education among adolescents (13-18 years old) from 1986 to 2026. The primary goal was to quantify the evolution of scholarly interest and identify the key collaborative structures defining the field. Specifically, this analysis aimed to evaluate trends in publication growth over the past four decades, identify shifts in research momentum, examine co-authorship networks to determine key research hubs and “bridge” authors connecting public health theory and clinical interventions, and analyze the thematic evolution of the field, with a focus on how educational strategies have developed within the literature. This approach was guided by established bibliometric methods for the medical literature, which support the use of quantitative publication data, authorship patterns, citation metrics, and network visualization to characterize research trends, collaborative structures, and thematic development within a defined field [10]. 

Eligibility Criteria

Studies were selected for inclusion based on a systematic keyword-based search strategy designed to capture the intersection of sun safety education and adolescent health. Articles were required to include terms related to the primary subject matter (“sun safety,” “sun protection,” or “skin cancer prevention”) in combination with terms identifying the target population (“adolescent,” “youth,” “high school,” or “students”) and the intervention type (“education,” “program,” “curriculum,” or “intervention”). To maintain the specific focus of this bibliometric analysis, the following inclusion criteria were applied: the search encompassed human-subject records indexed in PubMed, specifically targeting adolescents aged 13 to 18 years. To ensure consistent mapping of the research landscape, only English-language publications were analyzed. No specific restrictions were placed on study design or publication type; randomized controlled trials, observational studies, and various program evaluations were included to ensure a comprehensive representation of the collaborative network.

Information Sources

The sole data source for this publication was the PubMed database, maintained by the U.S. National Library of Medicine. The database was searched on April 6, 2026.

Search Strategy

**Figure 1:**
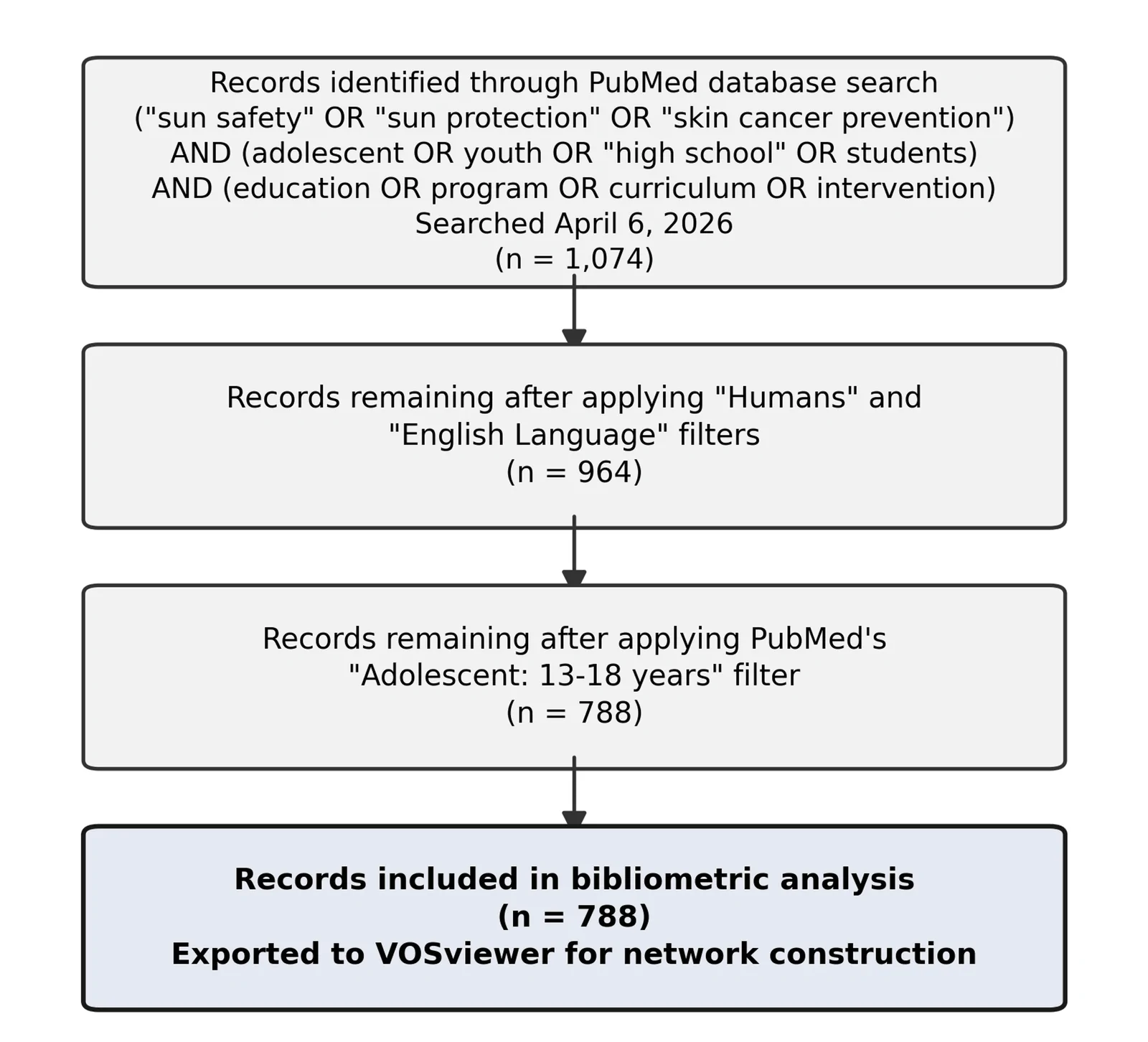
Flow diagram of record identification and selection The PubMed search identified 1,074 records. After applying the human-subject and English-language filters, 964 records remained. Application of PubMed’s “Adolescent: 13–18 years” filter yielded 788 records, all of which were included in the bibliometric analysis and exported to VOSviewer for network construction.

A keyword based search string was utilized using the following terms: ("sun safety" OR "sun protection" OR "skin cancer prevention") AND (adolescent OR youth OR "high school" OR students) AND (education OR program OR curriculum OR intervention). Filters were applied to restrict results to “Humans” and “English language”. To ensure the data specifically addressed the target demographic, PubMed’s "Adolescent: 13-18 years" filter was applied. It is important to note that while this filter captures studies focused on teenagers, these records often include comparative data or longitudinal follow-ups involving adult and middle-aged populations, which are reflected in the final keyword analysis. While no publication date restrictions were manually set, the resulting data set spanned from 1986 to 2026. The complete process of record identification and filtering is summarized in Figure 1. 

The complete PubMed search history, including field tags and PubMed's automatically mapped terms for each search element, is provided in the appendix.

Data Synthesis

All records identified through the search strategy were exported directly from PubMed in a format compatible with bibliometric software. In alignment with the study’s objectives to characterize the broad bibliometric landscape of sun safety education for adolescents, no manual screening or individual article exclusions were performed following data export. This absence of manual screening refers specifically to relevance screening and the exclusion of records from the analytic dataset; a separate, non-exclusionary classification of study design, described in the Results, was subsequently applied to a subset of records for descriptive purposes only and did not alter the composition of the dataset analyzed. The raw data were then imported into VOSviewer (Leiden University, the Netherlands) to construct and visualize the collaborative and thematic networks. 

Network maps were generated to analyze co-authorship patterns, institutional collaborations, and keyword co-occurrence relationships within the adolescent sun safety and skin cancer prevention literature. To ensure thematic depth of analysis, three distinct keyword networks were constructed: a general keyword co-occurrence map, a specialized author-keyword network, and a MeSH (Medical Subject Headings) term network. In addition to network visualizations produced in VOSviewer, descriptive statistics from PubMed were used to summarize publication trends from 1986 to 2026, characterizing the historical growth and shifting momentum of research dedicated to adolescent sun protection education.

Results

Article Types

Publication dates ranged from 1986 to 2026, with the majority of articles (n=713 of 788) published between 2001 and 2026. Over the past two decades (2000-2021), there was an increase in review articles (systematic and narrative, n=37), randomized controlled trials (n=107), cross-sectional studies (n=31, identified through manual extraction of study design described in abstracts), and meta-analyses (n=4), as well as a modest increase in case reports and case series (from n=1 prior to 2000 to n=5 between 2000 and 2021). Article types were classified based on study design as described in titles and abstracts. This classification was completed for the 2000-2021 period only and was not extended through 2026; article-type distribution for 2022-2026 was not assessed in this analysis.

Author Collaboration Network

**Figure 2:**
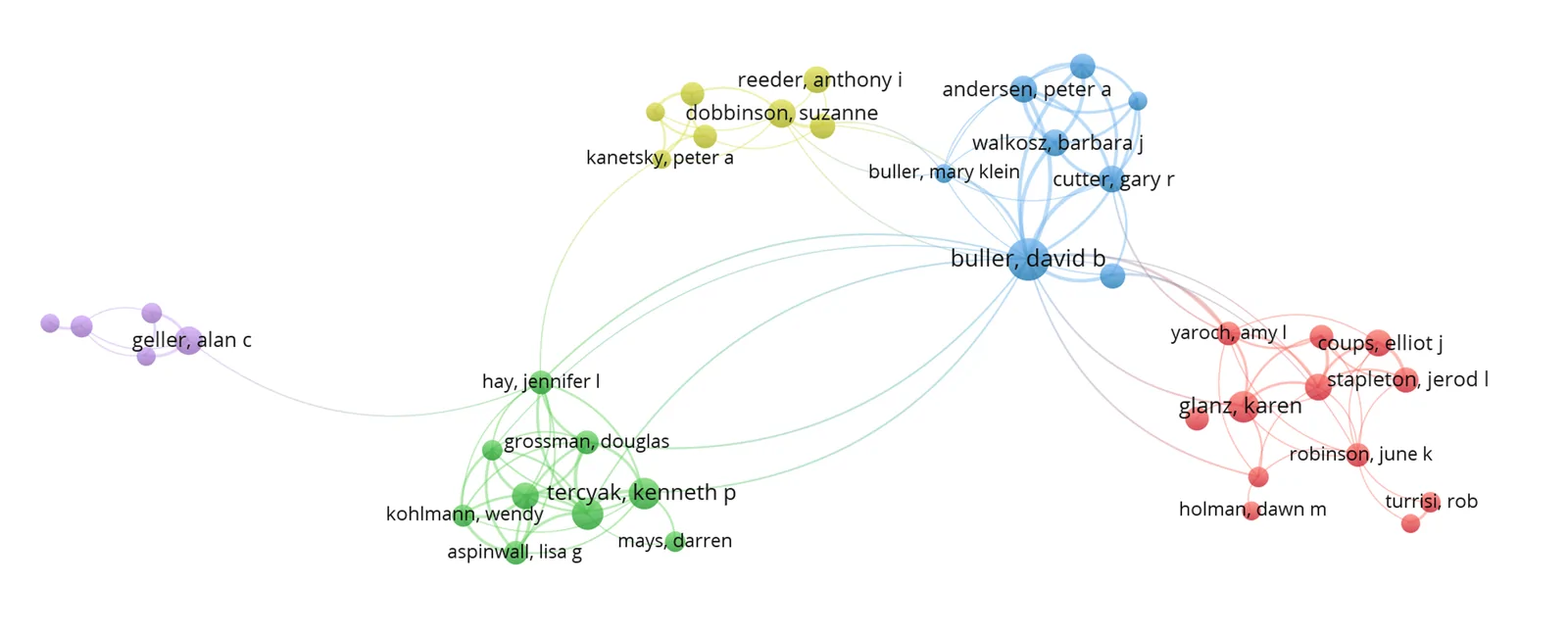
Co-authorship network of leading contributors to research on adolescent sun safety and skin cancer prevention education Visualization generated using VOSviewer (Leiden University, The Netherlands).

The co-authorship network revealed a series of interconnected thematic clusters rather than a single centralized core (Figure 2).

Buller DB represented the most prominent node, with a total link strength of 75, positioning him as the network’s primary anchor. The visualization revealed a methodological divide between public health theory (represented by the red cluster) and clinical research (represented by the green cluster). Specifically, the green cluster, led by Wu Y, represents a concentrated body of research dedicated to the 13-18-year-old demographic, focusing on the clinical and psychological aspects of sun safety within the adolescent population. The two clusters are spatially separated within the network map, with few direct co-authorship links observed between them in this visualization. Buller DB and Hay J occupy central positions connecting the surrounding clusters, consistent with a chain-like network structure.

Institutional Collaboration Network

**Figure 3:**
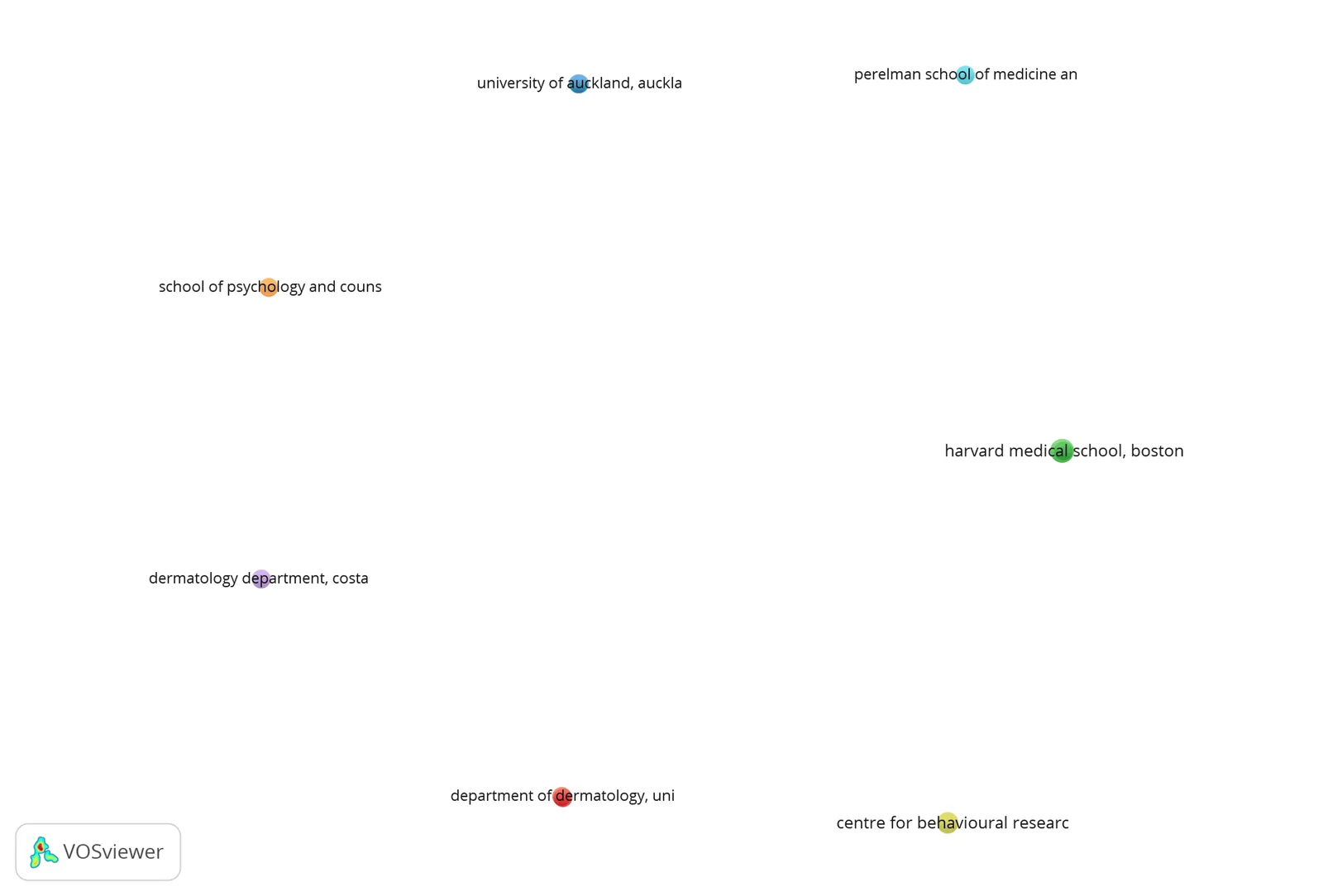
Institutional collaboration in research related to adolescent sun safety and skin cancer prevention education Visualization generated using VOSviewer (Leiden University, The Netherlands).

The institutional collaboration network identifies the primary academic hubs contributing to research on adolescent sun safety and skin cancer prevention (Figure 3).

Prominent institutions include Harvard Medical School, the Perelman School of Medicine at the University of Pennsylvania, and the University of Auckland. Notably, the visualization reveals a fragmented institutional landscape characterized by the absence of direct inter-institutional links among the leading organizations. Unlike the authorship network, which shows a chain of connectivity, the institutional map consists of isolated nodes spanning the United States and New Zealand, with no direct inter-institutional links observed in this visualization.

Keyword Co-occurrence Analysis (All Keywords)

**Figure 4:**
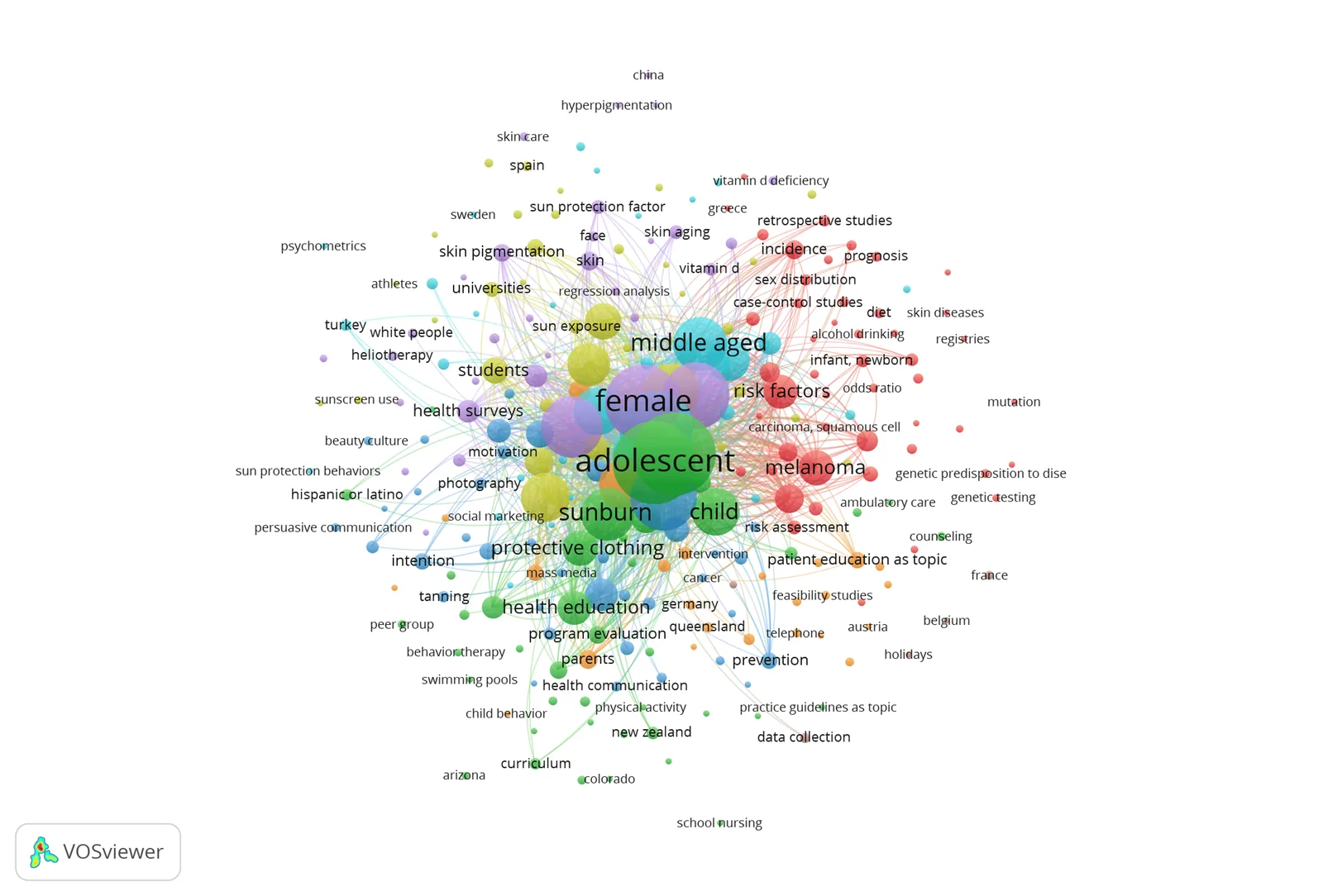
Keyword co-occurrence network in research related to adolescent sun safety and skin cancer prevention education Visualization generated using VOSviewer (Leiden University, The Netherlands).

The keyword co-occurrence analysis identifies a highly concentrated research core centered on “Adolescent,” “Skin Neoplasms,” “Adult,” “Middle Aged,” and “Health Knowledge” (Figure 4).

Although the study specifically filtered for the 13-18-year-old demographic, the prominence of the “Adult” and “Middle Aged” nodes indicates that adolescent sun safety is frequently studied within a longitudinal or life-course framework. This suggests that the literature characterizes sun-protective behaviors during adolescence as a primary preventive measure for reducing skin cancer risk later in life.

The mapping further reveals that “Health Education” and “Health Knowledge” serve as conceptual bridges between the target population and disease prevention. This core connection shows that the field remains heavily focused on the relationship between adolescents’ understanding of UV risks and their long-term health outcomes. Additionally, the analysis highlights a thematic cluster involving “Suntan,” “Sunbathing,” and “Sun Exposure,” reflecting the ongoing research focus on the tension between adolescent social norms and preventive compliance. While “Suntan” appears as a visible driver of this cluster, its proximity to “Sunburn,” often overlapping in high-density areas of the map, underscores the focus on the immediate physical consequences of UV exposure. Finally, the presence of terms such as “text messaging” and “feasibility studies” on the periphery suggests a modern shift in the literature toward testing digital, mobile-based interventions to reach the 13-18-year-old age group more effectively than older methods.

Author Keyword Co-occurrence Analysis

**Figure 5:**
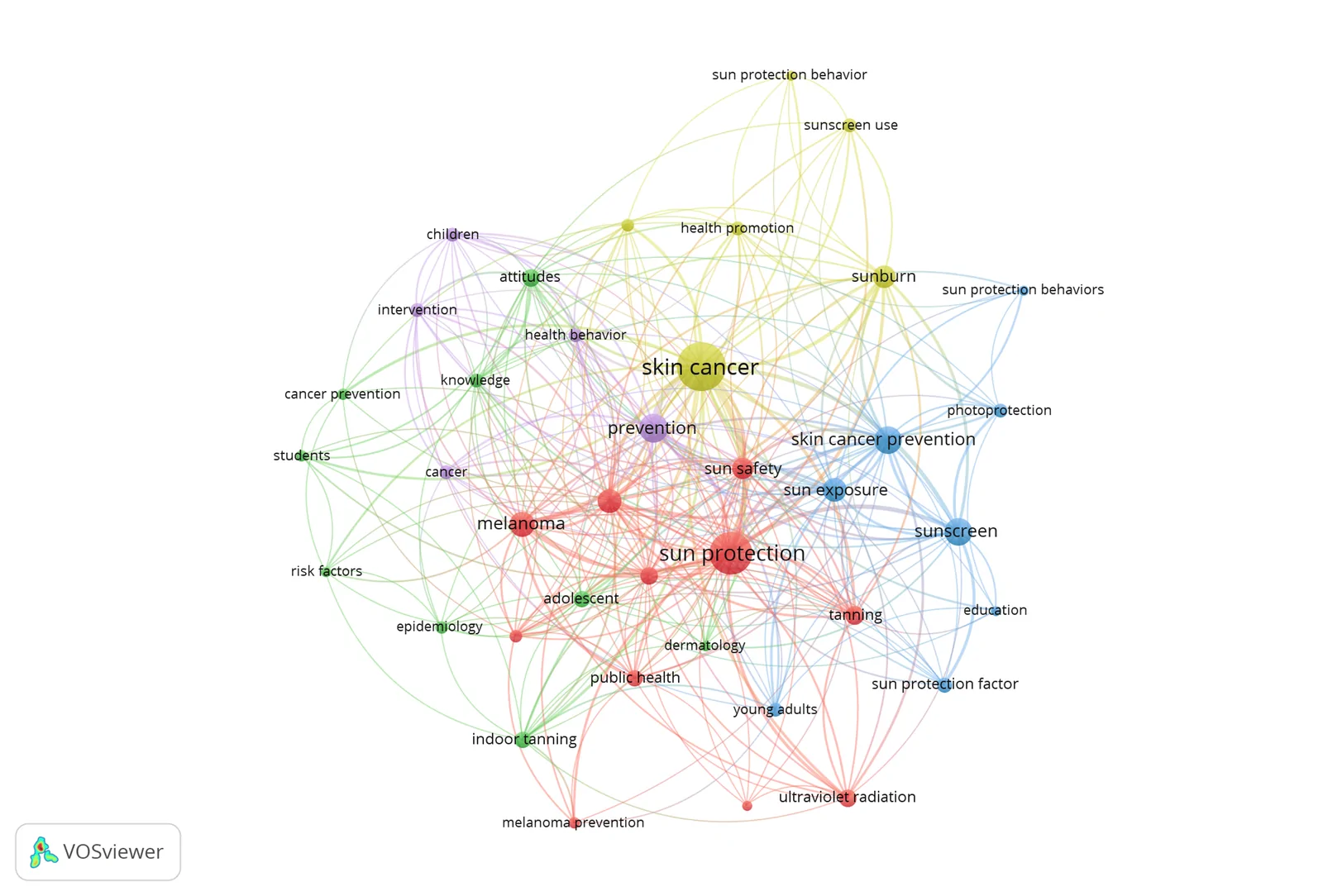
Author keyword co-occurrence network in research related to adolescent sun safety and skin cancer prevention education Visualization generated using VOSviewer (Leiden University, The Netherlands).

The author keyword co-occurrence network reveals the dominant research themes shaping the adolescent sun safety and skin cancer prevention literature (Figure 5).

“Skin cancer” and “sun protection” emerge as the central anchors of the field, forming a densely interconnected core alongside “melanoma,” “sunscreen,” and “skin cancer prevention,” reflecting research oriented predominantly toward clinical outcomes and protective products. In contrast, “health education” and “sun safety” appear with substantially lower frequency and connectivity, suggesting that educational implementation remains largely overlooked within the field’s primary research focus. The network also reveals an “indoor tanning” subcluster positioned at the epidemiologic periphery, suggesting that behavioral risk research has developed largely separately from education-focused research. Most critically, keywords related to health equity, such as “skin of color,” “Hispanic,” “African American,” and “ethnic groups,” were entirely absent from the network. This absence suggests that the unique sun safety needs and risk profiles of racially diverse adolescent populations have not been adequately addressed in the current literature.

MeSH Keyword Co-occurrence Network

**Figure 6:**
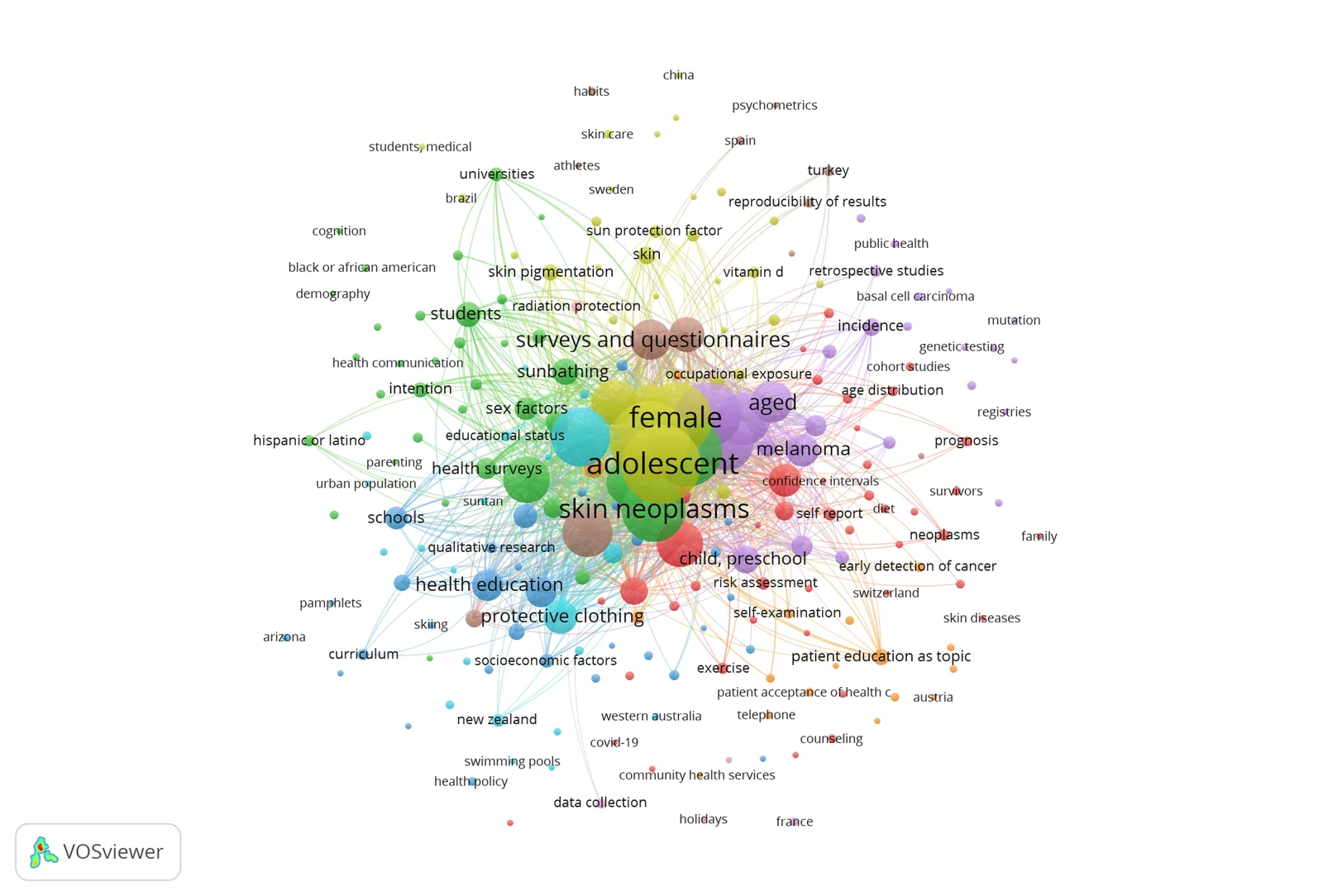
MeSH keyword co-occurrence network in research related to adolescent sun safety and skin cancer prevention education Visualization generated using VOSviewer (Leiden University, The Netherlands).

In the MeSH keyword co-occurrence network, nodes represent keywords selected by study authors and connecting lines indicate the frequency with which two keywords appear together within the same publication (Figure 6).

Analysis of author keywords identified several frequently occurring terms related to sun safety education and prevention among adolescents. The most common author keywords included “adolescent”, “skin neoplasms”, and “ultraviolet rays” reflecting the primary focus of the literature on youth populations and UV-related skin cancer risk. Additional keywords included “surveys and questionnaires,” “risk factors,” and “protective clothing,” displaying the strong emphasis on behavioral research and preventive strategies. The network also showed two main well-defined clusters: one focused on public health and epidemiology (“incidence,” “cohort studies,” “basal cell carcinoma”) and another focused on education and behavior (“schools,” “students,” “health promotion,” “intention”). These keywords highlight a significant gap in the literature: while adolescent knowledge and behaviors are well-documented, there is a comparative scarcity of studies evaluating the effectiveness of sun-safety interventions.

Publication Trends

**Figure 7:**
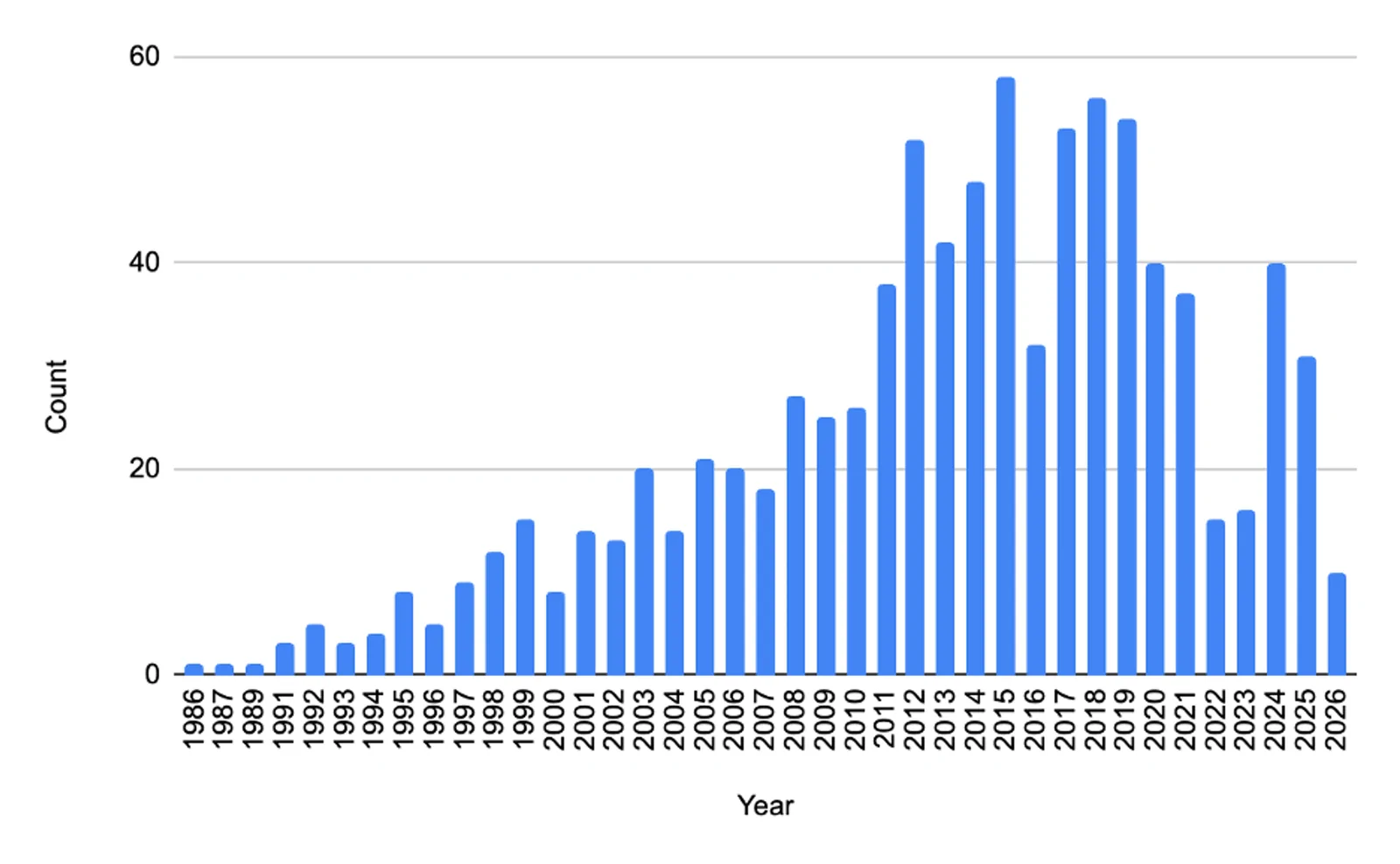
Annual distribution of publications on adolescent sun safety and skin cancer prevention education from 1985 to 2026

The chronological distribution of literature in the field shows a steady upward trajectory beginning in the early 1990s, with a significant surge in research around 2011 (Figure 7).

The volume of publications reached a historical peak in 2015, followed by a sustained period of high output between 2017 and 2019. While there was a visible decline in the most recent years (2025-2026), this is likely due to indexing delays and the inclusion of incomplete data for the current calendar year. This growth pattern reflects an intensifying global focus on adolescent sun safety and skin cancer prevention education, evolving from a niche area of study in the late 1980s into a robust, high-output research domain over the past decade.

Discussion

Analysis of publication trends, collaboration networks, and keyword patterns indicated that research on sun safety and skin cancer prevention had expanded substantially, driven by heightened public awareness, evolving prevention strategies, and broad global research output. The literature broadly reflected three phases of development: an early period focused on establishing foundational knowledge and raising awareness of ultraviolet (UV) exposure risks; a progressive phase characterized by the expansion and evaluation of sun safety education and prevention programs; and a more recent period marked by a surge in publication volume, a pivotal shift toward health equity and skin of color, and the integration of technology-driven tools, including digital health and artificial intelligence. These three phases reflect a narrative periodization of the literature based on qualitative review of publication content and trends over time, rather than a formal time-sliced keyword analysis, overlay visualization, or other quantitative temporal method. The following discussion presents a contextual narrative background on public health and cultural trends rather than findings derived directly from the co-authorship, institutional, or keyword networks described above.

Foundational Phase of Sun Safety Education (1986-2000)

Prior to 2000, sun safety and skin cancer prevention efforts were primarily introduced as large-scale, population-based public health initiatives. Early efforts gained momentum in the late 1980s with the launch of Australia's Slip! Slop! Slap! Campaign in 1981 and its subsequent formalization into the SunSmart program in 1988 [11]. In parallel, programs such as the U.S. Environmental Protection Agency (EPA) SunWise School Program in the United States, introduced in the late 1990s, expanded these efforts into school-based settings, emphasizing early education and behavioral awareness among children [12]. This period marked a transition toward structured, population-level prevention strategies, with clinicians and public health organizations increasingly implementing interventions aimed at reducing risk among high-exposure groups.

During this foundational period, interventions largely relied on broad public awareness campaigns, community-based education, and policy-driven approaches. Consequently, the resulting literature was primarily descriptive and epidemiological, focusing on population trends rather than the individualized and behavior-focused interventions that characterize more recent research. This early focus on prevention is further supported by clinical evidence, including a sibling case report of xeroderma pigmentosum that controlled for genetic and environmental factors and suggested that earlier initiation of sun protection independently delayed skin cancer onset and reduced disease severity, thereby reinforcing the importance of early-life UV protection [13].

Expansion of Epidemiologic and Behavioral Research (2001-2010)

The 2001-2010 period marked a transition from broad awareness toward the targeted epidemiologic characterization of adolescent UV risk behaviors. As publication volume grew, survey-based methodologies became dominant. Large cross-sectional datasets, most notably the Centers for Disease Control and Prevention (CDC)’s Youth Risk Behavior Surveillance System (YRBSS), facilitated national-level analyses of sunscreen use and adolescent health behaviors [14]. Findings from this era consistently highlighted a persistent “knowledge-behavior gap,” with studies reporting declining or inconsistent sunscreen use despite high levels of UV risk awareness [14,15].

This challenge was compounded by the rise of indoor tanning, which emerged as a major behavioral risk factor, particularly among White female adolescents. The literature demonstrated strong epidemiological associations between early tanning bed exposure and increased melanoma risk, prompting heightened public health concern and subsequent legislative action, including the 10% federal excise tax on indoor tanning services in 2010 [16]. Collectively, the expansion of behavioral research and the identification of high-risk practices shifted the field beyond descriptive awareness, setting the stage for the intervention-driven era that followed.

Rapid Growth and Shift Toward Intervention Strategies (2011-2026) 

In response to the compliance gap observed in the 13-18-year-old age group, the bibliometric record between 2011 and 2026 showed a decisive shift from observational studies toward the testing of active intervention modalities. Following a historical peak in publication volume in 2015, the literature began to prioritize multifaceted behavioral strategies that leveraged psychological frameworks to counteract the social capital of tanning [17]. This era is defined by an effort to move beyond simple knowledge sharing, instead focusing on interventions that address the “perceived rewards” of UV exposure versus the clinical “perceived costs” [18]. By engaging adolescents through digital autonomy rather than traditional methods, modern interventions, such as the use of UV photography to provide immediate visual feedback on skin damage, aimed to bridge the disconnect between health knowledge and actual behavior [19].

Despite the surge in technological innovation, the 2011-2026 literature revealed critical ongoing gaps in equity and institutional implementation. While research volume had increased, the bibliometric analysis highlighted a profound absence of keywords related to racially diverse populations, such as “Hispanic,” “African American,” or “skin of color.” This reflects a critical and persistent gap in addressing the unique risk profiles and delayed diagnosis patterns among adolescents with skin of color [20]. This gap is reflected in survey data showing that adolescents from racial and ethnic minority backgrounds and lower socioeconomic status scored lower on melanoma-knowledge assessments than their peers, despite facing disproportionate risk of delayed diagnosis [21].

This gap is reflected in survey data showing that adolescents from racial and ethnic minority backgrounds and lower socioeconomic status scored lower on melanoma-knowledge assessments than their peers, despite facing disproportionate risk of delayed diagnosis [21]. Additionally, the field faced a persistent “scalability gap,” in which successful digital pilots often struggled to transition into standardized, globally adopted health policies within school systems [22]. Consequently, while this peak era produced a wealth of innovative tools, the challenge of ensuring that these interventions are both inclusive and universally accessible remained [9].

Shifting Paradigms: Public Awareness, Policy, and the Skincare Culture

The following discussion presents contextual narrative background on public health and cultural trends rather than findings derived directly from the co-authorship, institutional, or keyword networks described above. The evolution of the research landscape is intricately linked to broader shifts in public health policy and cultural norms. Historically, the 1980s and 1990s were defined by a “tanning culture,” in which high-risk behaviors, such as the use of tanning accelerators and intentional rooftop sunbathing, were socially equated with health and status [15]. To combat these deep-seated norms, organizations such as the American Academy of Dermatology (AAD) and the Skin Cancer Foundation established large-scale public awareness campaigns that have since become institutionalized pillars of prevention. These efforts included the designation of May as Skin Cancer Awareness Month, anchored by “Melanoma Monday,” which served as a coordinated national public service announcement (PSA) campaign to promote early detection and sun-safe behaviors [23]. These initiatives transitioned skin cancer from a niche clinical concern to a mainstream public health priority, mirroring the success of awareness months for other major diseases.

More recently, a notable cultural shift has emerged, characterized by increased adolescent engagement with personal skincare regimens [24]. While earlier generations prioritized UV exposure for aesthetic appeal, the current 13-18-year-old demographic, particularly in higher socioeconomic regions, is increasingly invested in complex skincare regimens driven by social media trends. Observational and consumer health trends indicate that sunscreen is increasingly viewed by youth not merely as a seasonal medical precaution, but as a foundational daily product [25]. This trend offers an opportunity to align sun protection messaging with adolescents’ cosmetic motivations, including the prevention of photoaging, to improve long-term adherence beyond traditional risk-based counseling. Public health communicators and dermatology associations are increasingly leveraging these shifts through collaborative social marketing campaigns to promote daily sun protection factor (SPF) application as a routine health practice among youth [26].

Authorship Patterns and Collaborations

The authorship network exhibited high centrality among key investigators, with overall network connectivity anchored by central nodes such as Buller and Hay. While this provides a cohesive theoretical foundation, it also exposes a structural vulnerability: the field is reliant on individual relationships rather than systemic partnerships. The separation between public health theory (red cluster) and clinical researchers (green cluster) suggests that, while there is an understanding of how to change behavior and what the clinical risks are, these two groups rarely co-design interventions. To strengthen cross-field translation, highly connected nodes can serve as bridges to foster future interdisciplinary research linking behavioral theory with clinical dermatology.

Institutional Patterns and Collaborations: The Case for a Unified Front

The institutional landscape exhibits a decentralized structure, characterized by high-volume academic nodes operating with limited inter-institutional co-authorship. The limited co-authorship between major centers, such as Harvard and the University of Auckland may reflect geographic specificity of UV exposure risks and a primary focus on regional public health priorities [27]. Research efforts are often compartmentalized within national or regional borders because intervention strategies, such as school-based sun safety protocols, are frequently governed by local administrative mandates and varying state-level legislation [28]. However, cross-institutional datasets are increasingly essential for adolescent skin cancer prevention, as evaluating long-term behavioral outcomes requires longitudinal cohort sizes that single institutions rarely maintain. Establishing international collaborative networks could help standardized behavioral metrics for photoprotection and reduce duplication across localized pilot studies, ultimately strengthening the statistical power required to inform global public health policies. There is an urgent need for an international consortium to standardize metrics for “sun-safe behavior.” Without institutional collaboration, the field risks duplicating small-scale pilot studies that lack the statistical power to influence global health policy.

Adolescent Compliance Gap: Developmental and Social Dynamics

The 13-18-year-old demographic represents a narrow but disproportionately influential window during which long-term sun-protextion habits and health beliefs are frequently formed [29]. Bibliometric trends in this study highlight the challenge of addressing the shift from parental compliance to social autonomy. Children under 13 are largely compliant with sun safety practices because these behaviors are managed by their caregivers. However, as adolescents enter the 13-18-year-old age bracket, aesthetic motivations and the perceived link between tanning and attractiveness often override the known risks of UV exposure [30]. The keyword analysis indicated that while terms such as “Health Knowledge” maintained high occurrence, their co-occurence with “Suntan” and “Social Norms” underscored the strong influence of peer culture, illustrating that awareness alone may be insufficient to drive behavior adherence. This demographic presents unique intervention challenges, as adolescents frequently weigh immediate social rewards and aesthetic norms more heavily than long-term clinical outcomes, such as adult skin cancer risk. 

Keyword Analysis and Thematic Trends

The thematic shift from the 1980s to the present day reflects a broader revolution in health communication strategies. Early research (1986-1995) was dominated by passive educational tools such as posters, pamphlets, and school-based lectures [31]. The evolution of keywords indicates a transition from passive educational tools towards digital health interventions (DHIs). The emergence of terms such as “text messaging” and “feasibility studies” at the network periphery reflects growing interest in mobile-based engagement strategies for the 13-18-year-old demographic [32]. This pattern is corroborated by a 2025 review of mobile app-based sun protection interventions in pediatric populations, which found that while such tools are associated with short-term improvements in adolescents’ sun-protective intentions, the evidence base remains limited by self-reported outcomes, brief follow-up periods, and a lack of data on social determinants of use [33]. The emerging tools allow for real-time risk communication, such as automated UV index alerts, which may offer higher engagement than traditional static media in clinical settings. A key direction for future research is evaluating whether these emerging digital interventions can move beyond initial feasibility testing into validated, scalable public health tools.

Future Directions and Emerging Trends

The modern research landscape reflects a persistence knowledge-to-action gap among adolescents, where strong cognitive awareness of UV risks does not consistently translate into daily photoprotective behaviors [34]. Recent literature also highlights intersection of health equity considerations, showing that gender minority and transgender adolescents represent particularly vulnerable groups who report higher rates of indoor tanning compared to their cisgender peers [35]. Furthermore, as adolescents transition into their mid-teens, sun protection behaviors often decline. Longitudinal studies showed that the proportion of students who “often or always” use sunscreen when outdoors for extended periods dropped from 50% to just 25% over a three-year period [36].

Aesthetic motivations remained a significant psychological barrier to sun safety, with the social appeal of a tan often overriding health concerns. Adolescents who perceivde sun protection as inconvenient or socially unappealing remained less likely to adopt protective habits [17]. This barrier was consistently observed across prior research, which demonstrated that while primary prevention interventions reliably improved UV awareness, far fewer successfully drove sustained behavioral adjustments, such as routine shade-seeking or protective clothing use [37]. Even after recognizing the health risks, adolescents who perceivde tanning as enhancing their social appeal, reflecting the “perceived rewards” of UV exposure, remained largely unresponsive to risk-based messaging alone [38].

To bridge this gap, the focus is shifting toward specialized school-based programs and DHIs. Randomized trials have demonstrated that school-based curricula can successfully transition 30% of students toward more protective sun safety behaviors, specifically by improving their confidence in reading product labels and managing their own UV exposure [39]. Multi-institutional evaluations of peer-led outreach, such as the Sun Protection Outreach Teaching by Students (SPOTS) program, have demonstrated notable efficacy in modifying adolescent attitudes toward tanning and increasing intent to use sunscreen [40]. Additionally, digital health tools and mobile applications offer practical strategies to address adolescent forgetfulness through automated, UV index-triggered notifications [34,41].

Finally, the field is advancing toward “precision prevention,” which utilizes real-time physiological feedback and SMS-based action planning to motivate behavior change. Empirical models indicate that sun-protection self-efficacy serves as a far stronger determinant of actual behavior than general biological knowledge [36]. By incorporating appearance-focused strategies, such as UV photography that reveals existing sun damage, future interventions may reduce motivational barriers and empower adolescents to establish preventive habits that persist into adulthood [38].

This analysis was limited to English-language, human-subject records indexed in PubMed and identified using a relatively narrow search strategy. Consequently, relevant studies indexed in other databases or captured through broader terminology may have been omitted. Future bibliometric analyses should employ expanded search terms and a multidatabase approach to more comprehensively represent the interdisciplinary literature on adolescent sun safety. Additionally, this analysis does not report quantitative network metrics (e.g., node and link counts, total link strength, or clustering thresholds) for the co-authorship, institutional, or keyword networks, nor do the figure legends detail node size, link thickness, or inclusion thresholds; future work should report these parameters to improve reproducibility and interpretability of the network visualizations.

## Conclusions

This bibliometric review highlights the continued growth and evolution of research on adolescent sun safety and skin cancer prevention education. The findings suggest that the field has shifted from broad awareness efforts toward more targeted behavioral, school-based, and digital health interventions. However, opportunities remain to strengthen collaboration, improve consistency in outcome measurement, and expand research addressing racially and ethnically diverse skin phototypes. Future studies should prioritize scalable, inclusive, and developmentally appropriate strategies that promote lasting sun-protective behaviors among adolescents.

## Appendices

Appendix

Complete PubMed search history, field tags, automatic term mapping, and filters for: Global Trends in Sun Safety and Skin Cancer Prevention Education Among Adolescents: A Bibliometric Analysis

Search as Executed

Database: PubMed (U.S. National Library of Medicine)

Date searched: April 6, 2026

User-entered search string:

("sun safety" OR "sun protection" OR "skin cancer prevention") AND (adolescent OR youth OR "high school" OR students) AND (education OR program OR curriculum OR intervention)

Filters applied: English, Humans, Adolescent: 13-18 years

PubMed's Fully Translated Query

The fully translated query PubMed executed, as returned by PubMed's "Search Details" panel, was:

(("sun safety"[All Fields] OR "sun protection"[All Fields] OR "skin cancer prevention"[All Fields]) AND ("adolescences"[All Fields] OR "adolescency"[All Fields] OR "adolescent"[MeSH Terms] OR "adolescent"[All Fields] OR "adolescence"[All Fields] OR "adolescents"[All Fields] OR "adolescent's"[All Fields] OR ("adolescent"[MeSH Terms] OR "adolescent"[All Fields] OR "youth"[All Fields] OR "youths"[All Fields] OR "youth's"[All Fields]) OR "high school"[All Fields] OR ("student's"[All Fields] OR "students"[MeSH Terms] OR "students"[All Fields] OR "student"[All Fields] OR "students's"[All Fields])) AND ("educability"[All Fields] OR "educable"[All Fields] OR "educates"[All Fields] OR "education"[MeSH Subheading] OR "education"[All Fields] OR "educational status"[MeSH Terms] OR ("educational"[All Fields] AND "status"[All Fields]) OR "educational status"[All Fields] OR "education"[MeSH Terms] OR "education's"[All Fields] OR "educational"[All Fields] OR "educative"[All Fields] OR "educator"[All Fields] OR "educator's"[All Fields] OR "educators"[All Fields] OR "teaching"[MeSH Terms] OR "teaching"[All Fields] OR "educate"[All Fields] OR "educated"[All Fields] OR "educating"[All Fields] OR "educations"[All Fields] OR ("program"[All Fields] OR "program's"[All Fields] OR "programe"[All Fields] OR "programed"[All Fields] OR "programes"[All Fields] OR "programing"[All Fields] OR "programmability"[All Fields] OR "programmable"[All Fields] OR "programmably"[All Fields] OR "programme"[All Fields] OR "programme's"[All Fields] OR "programmed"[All Fields] OR "programmer"[All Fields] OR "programmer's"[All Fields] OR "programmers"[All Fields] OR "programmes"[All Fields] OR "programming"[All Fields] OR "programmings"[All Fields] OR "programs"[All Fields]) OR ("curriculum"[MeSH Terms] OR "curriculum"[All Fields] OR "curricula"[All Fields] OR "curriculums"[All Fields] OR "curriculum's"[All Fields] OR "education"[MeSH Subheading] OR "education"[All Fields]) OR ("intervention's"[All Fields] OR "interventions"[All Fields] OR "interventive"[All Fields] OR "methods"[MeSH Terms] OR "methods"[All Fields] OR "intervention"[All Fields] OR "interventional"[All Fields]))) AND ((humans[Filter]) AND (english[Filter]) AND (adolescent[Filter]))

Automatic Term Mapping (Per-Term Translations)

PubMed's Automatic Term Mapping (ATM) expanded each entered term as follows:

adolescent:

"adolescences"[All Fields] OR "adolescency"[All Fields] OR "adolescent"[MeSH Terms] OR "adolescent"[All Fields] OR "adolescence"[All Fields] OR "adolescents"[All Fields] OR "adolescent's"[All Fields]

youth:

"adolescent"[MeSH Terms] OR "adolescent"[All Fields] OR "youth"[All Fields] OR "youths"[All Fields] OR "youth's"[All Fields]

students:

"student's"[All Fields] OR "students"[MeSH Terms] OR "students"[All Fields] OR "student"[All Fields] OR "students's"[All Fields]

education:

"educability"[All Fields] OR "educable"[All Fields] OR "educates"[All Fields] OR "education"[Subheading] OR "education"[All Fields] OR "educational status"[MeSH Terms] OR ("educational"[All Fields] AND "status"[All Fields]) OR "educational status"[All Fields] OR "education"[MeSH Terms] OR "education's"[All Fields] OR "educational"[All Fields] OR "educative"[All Fields] OR "educator"[All Fields] OR "educator's"[All Fields] OR "educators"[All Fields] OR "teaching"[MeSH Terms] OR "teaching"[All Fields] OR "educate"[All Fields] OR "educated"[All Fields] OR "educating"[All Fields] OR "educations"[All Fields]

program:

"program"[All Fields] OR "program's"[All Fields] OR "programe"[All Fields] OR "programed"[All Fields] OR "programes"[All Fields] OR "programing"[All Fields] OR "programmability"[All Fields] OR "programmable"[All Fields] OR "programmably"[All Fields] OR "programme"[All Fields] OR "programme's"[All Fields] OR "programmed"[All Fields] OR "programmer"[All Fields] OR "programmer's"[All Fields] OR "programmers"[All Fields] OR "programmes"[All Fields] OR "programming"[All Fields] OR "programmings"[All Fields] OR "programs"[All Fields]

curriculum:

"curricula's"[All Fields] OR "curriculum"[MeSH Terms] OR "curriculum"[All Fields] OR "curricula"[All Fields] OR "curriculums"[All Fields] OR "curriculum's"[All Fields] OR "education"[Subheading] OR "education"[All Fields]

intervention:

"intervention's"[All Fields] OR "interventions"[All Fields] OR "interventive"[All Fields] OR "methods"[MeSH Terms] OR "methods"[All Fields] OR "intervention"[All Fields] OR "interventional"[All Fields]
